# Neurotoxicity of Brominated Flame Retardants: (In)direct Effects of Parent and Hydroxylated Polybrominated Diphenyl Ethers on the (Developing) Nervous System

**DOI:** 10.1289/ehp.1003035

**Published:** 2011-01-18

**Authors:** Milou M.L. Dingemans, Martin van den Berg, Remco H.S. Westerink

**Affiliations:** Neurotoxicology Research Group, Toxicology Division, Institute for Risk Assessment Sciences, Utrecht University, Utrecht, the Netherlands

**Keywords:** brominated flame retardant, calcium, developmental neurotoxicity, PBDE, persistent organic pollutant, polybrominated diphenyl ether, structure–activity relationship, thyroid

## Abstract

Background/objective: Polybrominated diphenyl ethers (PBDEs) and their hydroxylated (OH-) or methoxylated forms have been detected in humans. Because this raises concern about adverse effects on the developing brain, we reviewed the scientific literature on these mechanisms.

Data synthesis: Many rodent studies reported behavioral changes after developmental, neonatal, or adult exposure to PBDEs, and other studies documented subtle structural and functional alterations in brains of PBDE-exposed animals. Functional effects have been observed on synaptic plasticity and the glutamate–nitric oxide–cyclic guanosine monophosphate pathway. In the brain, changes have been observed in the expression of genes and proteins involved in synapse and axon formation, neuronal morphology, cell migration, synaptic plasticity, ion channels, and vesicular neurotransmitter release. Cellular and molecular mechanisms include effects on neuronal viability  (via apoptosis and oxidative stress), neuronal differentiation and migration, neurotransmitter release/uptake, neurotransmitter receptors and ion channels, calcium (Ca^2+^) homeostasis, and intracellular signaling pathways.

Discussion: Bioactivation of PBDEs by hydroxylation has been observed for several endocrine end points. This has also been observed for mechanisms related to neurodevelopment, including binding to thyroid hormone receptors and transport proteins, disruption of Ca^2+^ homeostasis, and modulation of GABA and nicotinic acetylcholine receptor function.

Conclusions: The increased hazard for developmental neurotoxicity by hydroxylated (OH-)PBDEs compared with their parent congeners via direct neurotoxicity and thyroid disruption clearly warrants further investigation into *a*) the role of oxidative metabolism in producing active metabolites of PBDEs and their impact on brain development; *b*) concentrations of parent and OH-PBDEs in the brain; and *c*) interactions between different environmental contaminants during exposure to mixtures, which may increase neurotoxicity.

Fire safety standards, including the application of flame-retardant chemicals, have been established in modern societies to reduce deaths and injuries as well as economic impact of fires. Bromine, like other halogens, quenches free radicals generated in a fire with high efficiency, thereby preventing the propagation of a flame. In Europe and North America, brominated flame retardants (BFRs) are therefore commonly used in household applications. BFRs were established as the major chemical flame retardant in the 1970s after the discovery of the adverse effects of polychlorinated biphenyls (PCBs) on human and environmental health and in experimental studies (for reviews, see Fonnum and Mariussen 2009; Seegal 1996). BFRs are a structurally diverse group of chemicals, with polybrominated diphenyl ethers (PBDEs) (Table 1) being among the formerly most highly produced BFRs (Bromine Science and Environmental Forum 2010).

**Table 1:** Individual PBDEs discussed in this review.

Table 1. Individual PBDEs discussed in this review.		
Abbreviation		Name
BDE-47		2,2´,4,4´-Tetrabrominated diphenyl ether
BDE-49		2,2´,4,5´-Tetrabrominated diphenyl ether
BDE-99		2,2´,4,4´,5-Pentabrominated diphenyl ether
BDE-100		2,2´,4,4´,6-Pentabrominated diphenyl ether
BDE-153		2,2´,4,4´,5,5´-Hexabrominated diphenyl ether
BDE-183		2,2´,3,4,4´,5´,6-Heptabrominated diphenyl ether
BDE-203		2,2´,3,4,4´,5,5´,6-Octabrominated diphenyl ether
BDE-206		2,2´,3,3´,4,4´,5,5´,6-Nonabrominated diphenyl ether
BDE-209		2,2´,3,3´,4,4´,5,5´,6,6´-Decabrominated diphenyl ether
DE-71		Commercial pentaBDE product
6-MeO-BDE-47		6-Methoxy-2,2´,4,4´-tetrabrominated diphenyl ether
6-OH-BDE-47		6-Hydroxy-2,2´,4,4´-tetrabrominated diphenyl ether

Although PBDEs are globally dispersed throughout the environment (reviewed by Law et al. 2008), human and environmental levels of PBDEs are approximately one order of magnitude higher in North America than in Europe and Asia (reviewed by Frederiksen et al. 2009). Humans are exposed to PBDEs in particular via air and ingestion of house dust, but also through intake of vegetable and animal products (reviewed by Frederiksen et al. 2009). Because of their relatively high food intake, child-specific hand-to-mouth behavior, and frequent ground contact (resulting in the ingestion of house dust), children are exposed to larger amounts of PBDEs than are adults (Fischer et al. 2006; Toms et al. 2009). An additional source of exposure for young children is breast milk, in which especially lower brominated PBDEs have been detected (reviewed by Frederiksen et al. 2009). Prenatal exposure to PBDEs occurs through placental transfer [Gómara et al. 2007; Mazdai et al. 2003; see Supplemental Material, Table 1 (doi:10.1289/ehp.1003035)].

Toxicokinetics studies have revealed the formation of methoxylated (MeO-) and hydroxylated (OH-)PBDEs *in vivo* (Malmberg et al. 2005; Staskal et al. 2006b; Wan et al. 2009; see also Supplemental Material (doi:10.1289/ehp.1003035)] and *in vitro* (Hamers et al. 2008; Stapleton et al. 2009; Wan et al. 2009). Interestingly, OH-PBDEs and MeO-PBDEs have also been detected as natural products in marine algae, cyanobacteria, and sponges (e.g., Malmvärn et al. 2008; Teuten et al. 2005). Human exposure studies have revealed that various OH-PBDEs are present in serum at concentrations similar to or sometimes higher than those of parent PBDEs (Athanasiadou et al. 2008; Qiu et al. 2009; see Supplemental Material, Table 1).

Since the start of this century, exposure to BFRs has been associated with (developmental) neurotoxicity (Eriksson et al. 2001). Recently, adverse effects on cognitive and neurodevelopmental parameters in humans were correlated to exposure to PBDEs. Motor, cognitive, and behavioral performance in 6-year-old Dutch children was correlated with maternal serum levels of PBDEs measured in the 35th week of pregnancy (Roze et al. 2009). In another study, the scores of U.S. children 0–6 years of age on yearly tests of mental and physical development were lower among those prenatally exposed to higher concentrations of PBDEs (Herbstman et al. 2010).

In this review we extend knowledge from previous reviews of underlying mechanisms of adverse effects on the nervous system by exposure to PBDEs (Costa and Giordano 2007; Fonnum and Mariussen 2009) and also focus on the contribution of OH-PBDEs to PBDE-induced neurotoxicity.

## Toxicity of PBDEs: Target Organs

Early acute *in vivo* toxicity studies have revealed only moderate effects of PBDEs at high exposure concentrations, mainly in the liver and thyroid gland (Hardy 2002). The first studies on the toxic mechanisms of PBDEs investigated the possible effects of PBDEs on aryl hydrocarbon receptor (AhR)-mediated processes because of their structural resemblance to AhR-activating compounds (e.g., some PCBs). Although effects of PBDEs were detected on gene induction and activity of hepatic enzymes *in vivo* and *in vitro* (van den Berg et al. 2006), the observed AhR-mediated effects of commercial PBDE mixtures are now generally considered to be caused by trace amounts of brominated dioxins and furans, which are potent inducers of cytochrome P450 (CYP) 1A and 1B enzymes (Sanders et al. 2005; van den Berg et al. 2006; Wahl et al. 2008). A lack of activation of the AhR by PBDEs, probably because of their lack of a planar-like structure, was recently confirmed in primary hepatocytes and hepatic cell lines (Wahl et al. 2010).

Endocrine and reproductive effects of PBDEs have been detected, in particular in wild birds and experimental studies with rodents (Dang et al. 2007; Fernie et al. 2008; Kuriyama et al. 2005). In epidemiological studies, exposure to PBDEs has been associated more recently with congenital cryptorchidism in Danish children (Main et al. 2007) and decreased fecundability (Harley et al. 2010).

*In vitro* studies have demonstrated that PBDEs and OH-PBDEs bind to estrogen, progesterone, androgen, and glucocorticoid receptors and inhibit CYP enzymes involved in steroidogenesis (Table 2) (Kojima et al. 2009; Meerts et al. 2001). Several studies have revealed that the potency of OH-PBDEs for these end points is higher than for their parent compounds, whereas MeO-PBDEs showed no or smaller effects (Table 2) (Cantón et al. 2005, 2006; Hamers et al. 2006; Mercado-Feliciano and Bigsby 2008).

**Table 2:** Effects and effect concentrations of BDE-47, 6-OH-BDE-47, and 6-MeO-BDE-47.

Table 2. Effects and effect concentrations of BDE-47, 6-OH-BDE-47, and 6-MeO-BDE-47.										
						Effect concentration (μM)				
Test system (study)		Effect		Exposure duration		BDE-47		6-OH-BDE-47		6-MeO-BDE-47
Human hepatoma (HepG2) cells (An et al. 2010)		Reduced cell viability		LOEC (72 hr)		—		0.5		5
		Cell cycle block		LOEC (48 hr)		—		0.5		NE (≤ 5)
		ROS generation		LOEC (24 hr)		—		0.1		2
Human adrenocortical carcinoma (H296R) cells (Cantón et al. 2005, 2006)		Reduced cell viability		LOEC (24 hr)		NE (≤ 7.5)		2.5		NE (≤ 7.5)
		Aromatase activity inhibition		LOEC (24 hr)		NE (≤ 7.5)		2.5		5
		CYP17 activity inhibition		LOEC (24 hr)		NE (≤ 10)		1		10
Human adrenocortical carcinoma (H296R) cells (Song et al. 2008)		Reduced cell viability		LOEC (48 hr)		NE (≤ 0.5)		NE (≤ 0.5)		NE (≤ 0.5)
		Aromatase activity inhibition		LOEC (48 hr)		NE (≤ 0.5)		NE (≤ 0.5)		NE (≤ 0.5)
Human placental microsomes (Cantón et al. 2008)		Aromatase activity inhibition		IC_50_ (40 min)		—		7.44		NE (≤ 40)
Nuclear hormone receptors transfected in CALUX cell lines (Hamers et al. 2006)		AhR		EC_50_ (24 hr)		NE (≤ 10)		1.3 ± 0.4		—
		AhR		IC_50_ (24 hr)		2.7 ± 0.7		NE (≤ 10)		—
		Androgen receptor		IC_50_ (24 hr)		1.0 ± 0.2		2.8 ± 0.4		—
		Progesterone receptor		IC_50_ (24 hr)		> 15		5.0 ± 0.9		—
		Estrogen receptor		EC_50_ (24 hr)		12 ± 4		NE (≤ 12.5)		—
		Estrogen receptor		IC_50_ (24 hr)		NE (≤ 12.5)		0.45 ± 0.03		—
Human TTR (Hamers et al. 2006)		TTR binding (T_4_ competition)		IC_50_ (binding equilibrium)		> 25		0.18		—
Human TTR (Marchesini et al. 2008)		TTR binding (T_4_ competition; biosensor)		IC_50_ (binding equilibrium)		NR (≤ 1)		0.087		NR (1.8)
Human TBG (Marchesini et al. 2008)		TBG binding (T_4_ competition; biosensor)		IC_50_ (binding equilibrium)		NR (≤ 10)		0.11		NR (10)
Zebrafish (van Boxtel et al. 2008)		Abnormal development		EC_50_ (72 hr)		NE (≤ 10)		0.025		NE (≤ 10)
		Mortality (adult)		EC_50_ (96 hr)		NE (≤ 0.5)		0.3		—
Neurotransmitter receptors expressed in *Xenopus* oocytes (Hendriks et al 2010)		GABA_A_-receptor full agonism		LOEC (20 sec)		NE (≤ 10)		10		—
		GABA_A_-receptor partial agonism		LOEC (10 sec)		NE (≤ 10)		10		—
		nACh-receptor antagonism		LOEC (5 sec)		NE (≤ 10)		10		—
Rat PC12 cells (Dingemans et al. 2010a)		Induction of [Ca^2+^]*i* oscillations		LOEC (20 min)		2		0.2		NE (≤ 20)
		Ca^2+^ release from intracellular stores		LOEC (20 min)		NE (≤ 20)		1		NE (≤ 20)
Abbreviations: —, not investigated; EC_50_, half-maximal effect concentration; GABA_A_, γ‑aminobutyric acid‑A; IC_50_, half-maximal inhibitory concentration; LOEC, lowest observed effect concentration; nACh, nicotinic acetylcholine; NE, no effect; NR, not reached; ROS, reactive oxygen species; TBG, T_4_-binding globulin.										

Epidemiological studies also showed correlations between human PBDE exposure and thyroid hormone levels (Chevrier et al. 2010; Turyk et al. 2008; Yuan et al. 2008). PBDEs and particularly OH-PBDEs structurally resemble the thyroid hormone thyroxin (T_4_) and have been found to bind to transthyretin (TTR), a thyroid hormone transport protein, and thyroid receptors α and β (Table 2) (Meerts et al. 2000). Effects of PBDEs and PBDE mixtures on the thyroid hormone system, mainly a decrease in T_4_ (hypothyroidism), were confirmed *in vivo* in rodents exposed prenatally and/or neonatally (Driscoll et al. 2009; Lee et al. 2010; Rice et al. 2007; Tseng et al. 2008; Zhou et al. 2001) or in adulthood (Darnerud et al. 2007; Richardson et al. 2008). Developmental exposure to low doses of BDE-47 (2,2´,4,4´-tetrabrominated diphenyl ether) resulted in changes in thyroid gland histology and morphology in rats (Talsness et al. 2008). In addition, a few studies found a reduction in the biologically active thyroid hormone triiodothyronine (T_3_) in serum of rats exposed to PBDEs (Lee et al. 2010; Tseng et al. 2008).

## Neurotoxicity of PBDEs: Behavioral Effects

Eriksson et al. (2001) exposed mice to a single oral dose of BDE-47 and BDE-99 (2,2´,4,4´,5-pentabrominated diphenyl ether) on postnatal day (PND) 10 and observed dose-dependent effects on spontaneous behavior (locomotion, rearing, and total activity) and Morris water maze relearning abilities of 2- and 4-month-old mice. Habituation capability also decreased with age in mice exposed to BDE-47 and BDE-99 compared with controls. This exposure regimen has been used to investigate effects of different PBDE congeners on spontaneous behavior. Similar long-lasting neurobehavioral changes have now been detected after a single oral dose of BDE-153 (2,2´,4,4´,5,5´-hexabrominated diphenyl ether), BDE-183 (2,2´,3,4,4´,5´,6-heptabrominated diphenyl ether), BDE-203 (2,2´,3,4,4´,5,5´,6-octabrominated diphenyl ether), BDE-206 (2,2´,3,3´,4,4´,5,5´,6-nonabrominated diphenyl ether), or BDE-209 (2,2´,3,3´,4,4´,5,5´,6,6´-decabrominated diphenyl ether) administered to neonatal mice (Johansson et al. 2008; Viberg et al. 2003, 2006). Further research has demonstrated effects of PBDEs on behavioral end points and performance in learning tasks in rodents after neonatal administration of a single oral dose, developmental exposure, or repeated neonatal dosing (Branchi et al. 2002; Dufault et al. 2005; Kuriyama et al. 2005). In a recent study, Cheng et al. (2009) reported impaired reflexes and Morris water maze relearning abilities in rats exposed to BDE-99 from gestation through lactation. Several developmental end points of motor activity and coordination were affected in mice exposed to a single oral dose of BDE-47 on PND10 (Gee and Moser 2008). By operant conditioning (reward-reinforced learning) of mice, increased impulsivity was observed in aging mice after neonatal exposure to BDE-209, whereas no effects were observed in littermates tested during young adulthood (Rice et al. 2009). In another study, rats were chronically exposed to the commercial pentaBDE mixture DE-71 during lactation and through feed, which was continued through testing, to mimic low-level chronic human exposure (Driscoll et al. 2009); no effects were observed on operant conditioning in adult rats, although their attention spans were impaired. This impaired attention is consistent with previously reported hyperactivity (i.e., increased locomotor activity) in animals postnatally exposed to various PBDEs (e.g., Eriksson et al. 2001), although it may also be related to the presence of brominated dioxins and furans in this commercial PBDE mixture.

The aforementioned studies strongly suggest that exposure to PBDEs causes adverse neurobehavioral effects, in particular during early neurodevelopment. With respect to neonatal exposure, a study in which BDE-99 was administered on PND3, PND10, or PND19 (Eriksson et al. 2002) revealed that brain development in mice is most sensitive to exposure to PBDEs in the first 2 weeks after birth. In rats and mice, rapid growth of the brain, as well as synaptogenesis and myelination, occurs within these 2 weeks, whereas in humans these processes start in the last trimester of pregnancy and extend into early childhood (for reviews, see Dobbing and Sands 1979; Rice and Barone 2000; Tau and Peterson 2010).

## Neurotoxicity of PBDEs: Effects on Brain Function and Structure

Using the same exposure regimen previously shown to affect neurobehavior (Eriksson et al. 2001), Dingemans et al. (2007) found that neonatal exposure to BDE-47 impaired long-term potentiation (LTP), a form of synaptic plasticity associated with learning and memory, measured *ex vivo* in hippocampal slices. Recently, Xing et al. (2009) reported that developmental exposure to the fully brominated BDE-209 also reduced LTP, as measured *in vivo* in the dentate gyrus of the hippocampus in rats. Rat offspring were exposed orally or via the mother to BDE-209 during different developmental periods (pregnancy, lactation, and after weaning). Decreases in synaptic plasticity were related to hippocampal BDE-209 concentrations, which were highest after intragastric exposure during the lactational period compared with after exposure via the mother (gestational or via lactation) or directly to the pups after weaning (Xing et al. 2009).

Developmental exposure [gestational days (GDs) 2–9] to BDE-99 resulted in increased activity of the glutamate–nitric oxide–cyclic guanosine monophosphate pathway in rat cerebellum (Llansola et al. 2007). This pathway is involved in the modulation of cerebral processes, including the release of neurotransmitters and synaptic plasticity (Boulton et al. 1995). Surprisingly, this increased activity coincided with a slightly enhanced performance in a learning task (Llansola et al. 2007).

Several studies have revealed effects of PBDEs on neurotransmitter receptor function and brain protein levels (Table 3). Effects of PBDEs on the cholinergic neurotransmitter system in rodents were revealed primarily by hypoactive responses to neurotransmitter receptor agonists (Johansson et al. 2008; Viberg et al. 2002). Particularly in hippocampus, but also in cortex and striatum, alterations in levels of key proteins involved in synaptic plasticity and brain development were detected after exposure to PBDEs (Alm et al. 2006; Dingemans et al. 2007; Viberg et al. 2008). Effects were observed after exposure to either tetra- and pentaBDEs or octa- through decaBDEs.  Potency differences between different PBDE congeners or degrees of bromination were not apparent.

**Table 3:** Effects of PBDE exposure on brain structure in rodents after oral administration during PNDs 1–14.

Table 3. Effects of PBDE exposure on brain structure in rodents after oral administration during PNDs 1–14.								
Species		Effect		PBDE		Effect concentration (μmol/kg body weight)		Reference
C57Bl/6 mouse		Decreased GluR subunit NR2B, GluR subunit GluR1, and p286-αCaMKII in hippocampal PSD (Western blot)		BDE-47		14		Dingemans et al. 2007
NMRI mouse		Decreased nAChR (decreased ^3^H-α-bungarotoxin binding)*a*		BDE-99		21		Viberg et al. 2004
NMRI mouse		Decreased nAChR (decreased ^3^H-α-bungarotoxin binding)*a*		BDE-99		14		Viberg et al. 2002
NMRI mouse		Alteration in hippocampal proteins (mainly proteins involved in metabolism and energy production, and in striatal proteins (mainly proteins involved in neurodegeneration and neuroplasticity; proteomics)		BDE-99		21		Alm et al. 2006
NMRI mouse		Alteration in cortical proteins (mainly cytoskeletal related; proteomics)		BDE-99		21		Alm et al. 2008
NMRI mouse		Decreased nAChR (decreased ^3^H-α-bungarotoxin binding)*a*		BDE-153		14		Viberg et al. 2003
NMRI mouse		Increased CaMKII and synaptophysin in hippocampus		BDE-203		21		Viberg 2009a
NMRI mouse		Increased CaMKII and synaptophysin in hippocampus		BDE-206		21		Viberg 2009a
Sprague-Dawley rat		Decreased nAChR (hypoactive *in vivo* nicotine response)*a*		BDE-209		21		Viberg et al. 2007
NMRI mouse		Decreased nAChR (hypoactive *in vivo* nicotine response)*a*		BDE-209		14		Johansson et al. 2008
NMRI mouse		Decreased BDNF and increased CaMKII and GAP-43 in hippocampus, decreased GAP-43 in cortex		BDE-209		21		Viberg et al. 2008
NMRI mouse		Increased synaptophysin in hippocampus		BDE-209		21		Viberg 2009b
Abbreviations: BDNF, brain-derived neurotrophic factor; CaMKII, Ca^2+^/calmodulin-dependent protein kinase II; GAP-43, growth-associated protein-43; GluR, glutamate receptor; nAChR, nACh receptor; p286-αCaMKII, autophosphorylated-active Ca^2+^/calmodulin-dependent protein kinase II; PSD, postsynaptic density. **a**Effects were detected on a whole-brain level.								

Bellés et al. (2010) detected markers for oxidative stress in the cerebellum and, to a lesser extent, in cortex and hippocampus of adult rats exposed to BDE-99 at 0.6 mg/kg body weight. In another study, Cheng et al. (2009) observed increased oxidative stress in the hippocampus (not in cerebellum and cerebral cortex) of rats exposed from GD6 to PND21 to BDE-99 at 2 mg/kg body weight via their mother (transplacental and lactational exposure). Only in this study (Cheng et al. 2009) were behavioral effects detected as well— perhaps related to the higher dose, but more likely related to the timing of the exposure (i.e., during neurodevelopment).

## Neurotoxicity of PBDEs: Cellular and Molecular Mechanisms

*Cell viability.* In several *in vitro* studies, tetra- and pentaBDEs were found to induce apoptotic cell death in primary neurons or neuronal cell lines, which appeared to be a consequence of oxidative stress (Giordano et al. 2008; He et al. 2008; Madia et al. 2004; Reistad et al. 2006; Yu et al. 2008). Recently, the fully brominated BDE-209 was also shown to increase apoptotic cell death and oxidative stress in rat primary hippocampal neurons (Chen et al. 2010). This effect may not be specifically neurotoxic, but neuronal cells are particularly vulnerable to oxidative stress because relatively high levels of reactive oxygen species (ROS) are generated during normal metabolism and neuronal activity. It appears that the antioxidant capacity of enzymes that are able to react with and inactivate ROS is exceeded after exposure to PBDEs, resulting in oxidative stress and subsequent damage of DNA, proteins, and membrane lipids.

*Cell differentiation and migration.* Differentiation and migration are critical processes in neurodevelopment. During *in vitro* exposure to BDE-209, neurite outgrowth and the differentiation of neural stem cells into neurons were dose-dependently inhibited [lowest observed effect concentration (LOEC), 10 μM]. Instead, a larger proportion of the neural stem cells differentiated into glial cells (Zhang et al. 2010). In another study, Schreiber et al. (2010) observed a decrease in the number of cells differentiating to neuron-like or oligodendrocyte-like cell types when human neural progenitor cells were exposed to 1 μM BDE-47 or BDE-99. Moreover, these *in vitro* experiments demonstrated decreased migration of human neural progenitor cells exposed to BDE-47 or BDE-99 (Schreiber et al. 2010), confirming previously suggested functional effects on cell migration, based on proteomics studies (Alm et al. 2008). In cultured fetal rat cortical cells, BDE-99–induced changes were also detected in levels of proteins involved in formation of the cytoskeleton that is involved in cellular migration (Alm et al. 2010).

*Neuronal signaling.* PBDEs (mainly tetra- and pentaBDEs) have been shown to affect several aspects of inter- and intraneuronal signaling, including inhibition of neurotransmitter uptake in synaptosomes and neurotransmitter vesicles and alterations in calcium (Ca^2+^) homeostasis (Kodavanti and Ward 2005; Mariussen and Fonnum 2003). DE-71 has recently been shown to reduce synaptosomal dopamine levels and increase medium dopamine concentrations in a system with isolated rat striatal synaptosomes, probably by inhibition of the membrane dopamine transporter (Dreiem et al. 2010). In addition to effects on neurotransmitter uptake, BDE-47 (20 μM) induced vesicular neurotransmitter release by exocytosis in PC12 pheochromocytoma cells (Dingemans et al. 2007). Importantly, the BDE-47 metabolite 6-OH-BDE-47 (5 μM) induced a more pronounced increase in exocytosis in PC12 cells within several minutes after the start of exposure (Dingemans et al. 2008). The observed increases in exocytosis were paralleled by increases in intracellular calcium concentration ([Ca^2+^]*_i_*). At 2 μM, BDE-47 induced only subtle changes in Ca^2+^ homeostasis that manifested as increased frequency and duration of fluctuations in [Ca^2+^]*_i_*, whereas no effects were observed on [Ca^2+^]*_i_* when PC12 cells were exposed to BDE-49 (2,2´,4,5´-tetrabrominated diphenyl ether), BDE-99, BDE-100 (2,2´,4,4´,6-pentabrominated diphenyl ether), or BDE-153 (Dingemans et al. 2010a). On the other hand, 6-OH-BDE-47 (6-hydroxy-2,2´,4,4´-tetrabrominated diphenyl ether; ≥ 1 μM) caused a rapid, robust increase in [Ca^2+^]*_i_* by release from endoplasmic reticulum followed by release from mitochondria. Similar effects were detected for other OH-PBDEs [6-MeO-BDE-47 (6-methoxy-2,2´,4,4´-tetrabrominated diphenyl ether) showed no effect]; although the potency to disturb calcium homeostasis depended on the presence of large atomic groups (bromine atom or aromatic ring) adjacent to the OH, the hydroxylation position was of lesser importance (Dingemans et al. 2010a). Although release of calcium from intracellular stores was not detected during exposure to parent PBDEs, DE-71, BDE-47, and BDE-99 have been shown to inhibit uptake of Ca^2+^ in microsomes and mitochondria isolated from rat cortex, cerebellum, and hippocampus (Coburn et al. 2008). BDE-47 and OH-PBDEs that increased basal [Ca^2+^]*_i_* subsequently inhibited the depolarization-evoked increase in [Ca^2+^]*_i_* in PC12 cells at similar LOECs (Dingemans et al. 2010b). Although this appeared to be a direct inhibitory effect on calcium entry, it was strongly potentiated by preceding increases in basal [Ca^2+^]*_i_* before depolarization, suggesting additional Ca^2+^-dependent regulatory mechanisms (Dingemans et al. 2010b).

PBDEs have also been shown to affect several aspects of intracellular signaling pathways that are involved in Ca^2+^ homeostasis and Ca^2+^ signaling (García et al. 2006) by inducing protein kinase C translocation and arachidonic acid release via phospholipase A activation (Kodavanti and Derr-Yellin 2002; Kodavanti and Ward 2005; Madia et al. 2004). In cerebellar granule neurons, PBDE congeners and commercial mixtures (low micromolar range) also activated the mitogen-activated protein kinase pathway (Fan et al. 2010), which is involved in the modulation of various cellular functions and in turn is modulated by both protein kinase C and Ca^2+^ (Pearson et al. 2001; Schönwasser et al. 1998).

BDE-209 dose-dependently decreased voltage-gated sodium channel currents in primary rat hippocampal neurons (Xing et al. 2010). Recently, full and partial agonistic effects on the γ-aminobutyric acid-A (GABA_A_) receptor and antagonistic effects on the α_4_β_2_ nicotinic acetylcholine (nACh) receptor (expressed in *Xenopus* oocytes) were demonstrated for 6-OH-BDE-47, whereas these effects were not observed for BDE-47 (Hendriks et al. 2010). Because interactions of GABAergic, cholinergic, and glutaminergic neurotransmitters are critical for the development of neuronal networks during brain development (Ben-Ari et al. 2007), these opposite effects on excitatory (nACh) and inhibitory (GABA) neurotransmitter receptor effects suggest an important role of the OH-PBDEs in the observed *in vivo* neurotoxicity of PBDEs.

Together, these *in vitro* studies indicate that lower brominated PBDEs (tetra- and pentaBDEs) affect all levels of neurotransmission, from presynaptic neurotransmitter homeostasis, intracellular signaling, and neurotransmitter release to postsynaptic neurotransmitter receptors. For some neurotoxic end points, it is evident that OH-PBDEs have a higher potency than their parent congeners (Table 2), whereas this is unclear for other end points. Because intracellular signaling pathways, calcium homeostasis, and oxidative stress are interrelated, the main molecular target of (OH-)PBDEs remains unclear. However, the combination of results from several studies showing oxidative stress or mitochondrial Ca^2+^ release (or inhibition of Ca^2+^ uptake) suggests that mitochondria are a primary target for PBDE toxicity. This is supported by the observation of accumulation of PBDEs in mitochondria in mouse primary neurons (Huang et al. 2010). A combination of the observed cellular and molecular mechanisms could be involved in the observed neurobehavioral impairments. In particular, OH-PBDEs have been shown to alter neurotransmitter release patterns and underlying (Ca^2+^) signaling pathways. Influence of transient alterations in [Ca^2+^]*_i_* on brain development is not unlikely, because Ca^2+^ signals are essential in (early) brain development (for reviews, see Moody and Bosma 2005; Spitzer 2006) and the induction of synaptic plasticity (for reviews, see Malenka and Nicoll 1999; Soderling and Derkach 2000). Small changes in catecholaminergic modulation can have effects on behavior (Arnsten and Li 2005). Temporary alterations of neuronal activity during critical periods in brain development could therefore underlie the observed effects on behavior and hippocampal synaptic plasticity. The concentrations at which effects of parent PBDEs are observed *in vitro* are very different from human exposure levels. However, few *in vitro* studies take into account the low solubility of PBDEs or the binding of PBDEs to serum proteins and cell culture materials, resulting in overestimation of cellular exposure concentrations. In contrast, accumulation into cells has also been demonstrated to occur, resulting in the possible underestimation of intracellular PBDE concentrations after (sub)chronic exposure (Huang et al. 2010; Mundy et al. 2004).

## Conclusion and Discussion

**Figure 1:**
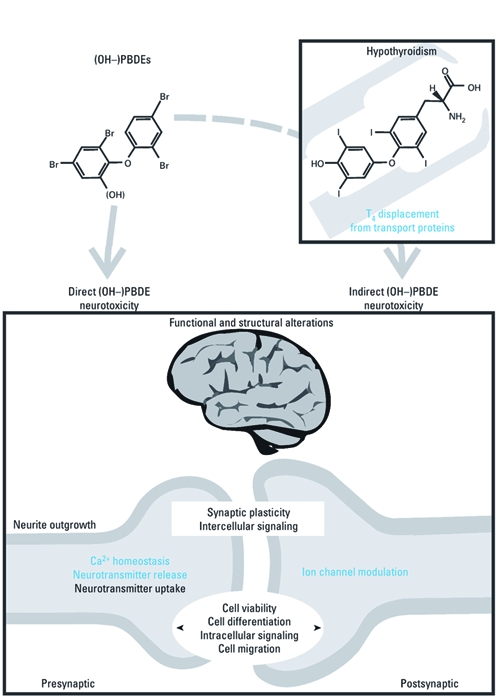
Direct and indirect PBDE-induced developmental neurotoxicity. Observed end points for which larger effects have been detected for OH-PBDEs are shown in blue.

Behavioral studies clearly indicate the neurotoxic potential of PBDEs. Importantly, several mechanistic studies indicate that this neurotoxic potential is higher for the OH-PBDEs. At least two effects of PBDEs or their metabolites are expected to be involved in developmental neurotoxicity, resulting in structural and functional alterations in the brain and, ultimately, behavioral effects (Figure 1). On the one hand, direct toxic effects on the (developing) nervous system are likely involved in PBDE-induced developmental neurotoxicity. On the other hand, PBDEs have been shown to affect the thyroid hormone system. Because thyroid hormones are critically involved in (early) brain development (for reviews, see Horn and Heuer 2010; Howdeshell 2002), this mechanism is likely involved in the PBDE-induced neurotoxicity.

In humans, thyroid hormone production starts at approximately 10 weeks of gestation and increases with the development of the fetal hypothalamic–pituitary–thyroid axis (reviewed by Howdeshell 2002). However, transplacental transfer of maternal thyroid hormones to the fetus is critical for neurodevelopment. Impaired psychomotor development and visuospatial processing have been revealed in children born to mothers with (subclinical) hypothyroidism (Haddow et al. 1999; Pop et al. 1999). Prenatal and postnatal hypothyroidism are associated with behavioral deficits in humans as well as animal models (reviewed by Zoeller and Rovet 2004).

On a cellular level, effects of thyroid hormones have been shown to be involved in neuronal proliferation, migration, synaptogenesis, synaptic plasticity, and myelation processes (for reviews, see Horn and Heuer 2010; Howdeshell 2002). Interestingly, several of these processes (described above) have been shown to be directly affected by PBDEs or their metabolites, and adverse effects comparable to those observed after developmental exposure to BFRs are also observed in animal models of moderate or transient insufficiency in thyroid hormone levels, commonly induced by exposure to propylthiouracil (reviewed by Zoeller and Crofton 2005).

*In vitro* studies with 6-OH-BDE-47 show that effect concentrations for displacement of T_4_ from the hormone transport protein TTR and induction of [Ca^2+^]*_i_* oscillations are within the same order of magnitude (~ 0.2 μM; Table 2). Several animal studies have simultaneously investigated both PBDE-induced neurotoxicity and effects on thyroid hormone levels. In rodents in which neonatal exposure to DE-71 and BDE-209 caused effects on neurobehavior, hypothyroidism was also observed (Driscoll et al. 2009; Rice et al. 2007). Experimentally induced hypothyroidism has been shown to result in a decrease in cholinergic nerve endings (Sawin et al. 1998). Therefore, the disruption of normal brain development by (OH-)PBDE-induced hypothyroidism may be an underlying mechanism for the observed PBDE-induced alterations in the cholinergic neurotransmitter system in rodents. However, other mechanisms are likely involved, because thyroid hormone levels were not affected in mice exposed at PND10 to a single oral dose of BDE-47, which is known to result in behavioral effects (Gee et al. 2008). On the other hand, for rat dams and offspring with very low levels of T_4_ after developmental exposure to DE-71, an effect was detected in only one of several behavioral tests (Kodavanti et al. 2010). This indicates that other mechanisms are also involved in PBDE-induced behavioral neurotoxicity, and that these may differ for various (behavioral) neurotoxic end points and timing of exposure. Further investigation is required to determine the relative contributions or possible effect additivity of the direct and indirect (via thyroid disruption) neurotoxic effects of (OH-)PBDEs in humans. Future studies on thyroid disruption also have to take into account that thyroid hormone levels within brain tissue are more informative of possible thyroid-related effects, because cellular actions of thyroid hormones are initiated at nuclear receptors (reviewed by Cheng et al. 2010).

The binding of PBDEs and especially OH-PBDEs to receptors and transport proteins involved in thyroid homeostasis and function is the expected mode of action of PBDE-induced hypothyroidism (Marchesini et al. 2008; Meerts et al. 2000; Schriks et al. 2006; Ucán-Marín et al. 2009). However, other interactions with the thyroid system, for example, inhibition of iodine uptake such as observed for perchlorate (reviewed by Jugan et al. 2010), are also possible and require further study. It has been revealed only recently that BDE-209 inhibits thyroid-hormone–induced dendrite arborization of rat Purkinje cells, probably via partial dissociation of thyroid receptors from the binding domain on the thyroid-receptor–responsive element (Ibhazehiebo et al. 2010). It is commonly assumed that up-regulation of thyroid- stimulating hormone and deiodinase activity in the brain compensates for reductions in brain T_4_ levels. However, it has recently been revealed that compensatory mechanisms do not prevent effects on thyroid hormone targets in the brain resulting from small reductions in serum T_4_ (Sharlin et al. 2010).

Various environmental pollutants can disrupt thyroid homeostasis (reviewed by Jugan et al. 2010). Especially if effects on thyroid homeostasis coincide with direct neurotoxicity, as observed for PBDEs, developmental neurotoxicity has to be anticipated. Iodine deficiency, a common condition worldwide (reviewed by Walker et al. 2007), is an additional risk factor to increase the sensitivity to adverse effects from thyroid-disrupting chemicals.

Because of the strict temporal regulation of brain development processes, certain time windows are particularly sensitive to neurotoxic insults. Although brain development in humans extends into early childhood, the perinatal period of brain development (“brain growth spurt”; last trimester to 1–2 years of age) has especially been proven to be very sensitive to neurotoxic effects (reviewed by Rice and Barone 2000). It is currently difficult to estimate whether gestational or lactational exposure is most relevant for (OH-)PBDE-induced developmental neurotoxicity in humans. The lower exposure during a sensitive (possibly prenatal) developmental phase may be more hazardous for the developing fetal brain than higher exposure during the next (possibly postnatal) developmental phase via for example, lactational transfer. Further research on neurodevelopment and cognitive and behavioral function, in particularly with respect to disruption by external stimuli, including xenobiotics, is therefore required.

Partitioning of PBDEs into the (fetal) brain is detected in wildlife (Basu et al. 2007; Gebbink et al. 2008; Montie et al. 2009) and toxicokinetics studies, often by using radiolabeled PBDEs (Örn and Klasson-Wehler 1998; Riu et al. 2008; Staskal et al. 2006a, 2006b). In contrast, recent experimental and wildlife studies have shown that OH-PBDEs are usually not detectable in brain, although OH-PBDEs were detected in brain and cerebrospinal fluids of marine mammals (Gebbink et al. 2008; Montie et al. 2009; Zhang et al. 2008). Nonetheless, it should be recognized that for some OH-PCBs, specific accumulation in brain tissue occurs, which has been suggested to be associated with binding to TTR (Meerts et al. 2002; Morse et al. 1996). OH-PBDEs also strongly bind to TTR as well as to T_4_-binding globulin (TBG), which is the main transporter protein for thyroid hormones in humans across the blood–brain barrier (Marchesini et al. 2008). Because of the discrepancy between observations in wildlife and toxicokinetics studies with parent PBDEs, as well as the demonstrated accumulating properties of the structurally related (OH-)PCBs, the potential of OH-PBDEs to enter the brain requires further study.

For most classes of chemicals, risk assessment is based on exposure and hazard assessment of single compounds, with the exception of the toxic equivalency approach for dioxins and dioxin-like compounds (van den Berg et al. 2006). In reality, however, the human exposure pattern includes a plethora of chemicals, including environmental and dietary (OH-)PBDE exposure and those originating from systemic metabolic conversion. For the latter aspect, interindividual differences commonly occur (Hong and Yang 1997). Because of the observed similarities in effects on neurobehavior and on acute *in vitro* effects related to neuronal signaling, there is a scientific rationale to use concentration addition as the default precautionary approach for risk assessment of mixtures of PBDEs, PCBs, and their metabolites. Because of the possible additivity of these environmental pollutants at levels below effective concentrations of the individual congeners and because of the higher sensitivity of the developing brain, mixture effects must be taken into account for human risk assessment for developmental neurotoxicity and investigated further.

Although the use of PBDEs has been greatly reduced in most countries because of voluntary and legislative measures, humans and wildlife will still be exposed to PBDEs and their metabolites for many years to come because of their environmental persistence and the fact that consumer products with these flame retardants are still present in many homes. This also underscores the necessity of implementing better hazard and risk assessment strategies for identifying human health risks of new chemicals, such as novel halogenated flame retardants. More knowledge on oxidative metabolism and the possible bioactivation of PBDEs, the sensitivity to neurotoxicity during specific processes of brain development, the concentrations of (OH-)PBDEs in the brain, and the interactions between different environmental contaminants during simultaneous exposure is currently required to further improve the assessment of neurotoxic risk of PBDEs, their metabolites, and related environmental contaminants.

## Supplemental Material

(148 KB) PDF
