# Supplementary material for: Neurotoxicity of Brominated Flame Retardants: (In)direct Effects of Parent and Hydroxylated Polybrominated Diphenyl Ethers on the (Developing) Nervous System
Source: Environ Health Perspect. 2011 Jan 18;119(7):900–7. doi: 10.1289/ehp.1003035 (PMC3223008; doi:10.1289/ehp.1003035)
Supplement: (148 KB) PDF [file ehp.1003035.s001.pdf]

Supplemental Material

Neurotoxicity of Brominated Flame Retardants: (In-)Direct Effects of Parent and Hydroxylated Polybrominated Diphenyl Ethers on the (Developing) Nervous System

Milou ML Dingemans[1]*, Martin van den Berg[1], Remco HS Westerink[1]

[1] Neurotoxicology Research Group, Toxicology Division, Institute for Risk Assessment Sciences, Utrecht University, Utrecht, the Netherlands

Table of Contents

Supplemental Material, Title page 1

Supplemental Material, Table 1. Human serum levels of PBDEs and PBDE metabolites 2

Supplemental Material, Toxicokinetics of PBDEs and OH-PBDEs 3

Supplemental Material, References 6

Abbreviations supplemental

n.a.	not applicable
n.d.	not detected
OH-PBDE	hydroxylated polybrominated diphenyl ether
PBDE	polybrominated diphenyl ether

Supplemental Material, Table 1. Human serum levels of PBDEs and PBDE metabolites[a].

sample	PBDEs	median (range; ng/g lipids)	references
18-months old, US	BDE-47	n.a. (245)	
	ΣPBDE[b]	n.a. (651)	Fischer et al. 2006
2-6 year olds, Australia	ΣPBDE	41 (33 - 49)	Toms et al. 2009
adults, the Netherlands	BDE-47	0.8 (0.1 - 6.1)	
	ΣPBDE	3.3 (0.5 - 33.1)	
	6-OH-BDE-47	n.d. (n.d.)	Meijer et al. 2008
adults, Sweden	BDE-47	3.5 (n.d. - 8.3)	
	ΣPBDE[b]	20.3 (8.6 - 50.5)	Karlsson et al. 2007
adults, Spain	BDE-47	2.4 (0.3 - 9)	
	ΣPBDE[b]	12 (5.5 - 43)	Ramos et al. 2007
adults, South-China	BDE-47	1.0 (0.4 - 3.6)	
	ΣPBDE	4.4 (1.6 - 17)	Bi et al. 2006
adults, US	BDE-47	10 (<10 - 511)	Petreas et al. 2003
adults, US	BDE-47	28 (9.2 - 310)	
	ΣPBDE	37 (15 - 580)	Mazdai et al. 2003
adults, US	BDE-47	15.2 (8.0 - 28.9)	
	ΣPBDE	34.0 (17.9 - 50.8)	
	6-OH-BDE-47	0.3 (0.1 - 0.5)	
	ΣOH-PBDE	6.3 (3.8 - 11.3)	Qiu et al. 2009
adults, Korea	6-OH-BDE-47	<4 (<4 – 177)[c]	Wan et al. 2010
cord blood, South-China	BDE-47	1.4 (0.1 - 4.9)	
	ΣPBDE	3.9 (1.5 - 12)	Bi et al. 2006
cord blood, Spain	BDE-47	3.3 (<0.1 - 35)	
	ΣPBDE[b]	17 (6.3 - 82)	Gómara et al. 2007
cord blood, Sweden	BDE-47	0.98 (0.3 - 3.3)	
	ΣPBDE	1.7 (0.5 - 4.3)	Guvenius et al. 2003
cord blood, the Netherlands	6-OH-BDE-47	n.d. (n.d.)	Meijer et al. 2008
cord blood, US	BDE-47	25 (8.4 - 210)	
	ΣPBDE	39 (14 - 460)	Mazdai et al. 2003
cord blood, US	BDE-47	13.45 (2.6 - 550.9)	
	ΣPBDE	30.9 (4.7 - 797.6)	
	6-OH-BDE-47	1.0 (0.1 - 62.1)	
	ΣOH-PBDE	22.0 (2.0 - 899.1)	Qiu et al. 2009
cord blood, Korea	6-OH-BDE-47	26 (<4 – 127)[c]	Wan et al. 2010
electronics dismantlers, US	BDE-47	4.8 (<0.5 - 23.4)	
	ΣPBDE[b]	26 (7.5 - 37.3)	Sjödin et al. 1999
electronics dismantlers, Norway	BDE-47	4.0 (mean; 0.9 - 15)	
	ΣPBDE	8.8 (mean; 3.8 - 24)	Thomsen et al. 2001
computer technicians, Sweden	BDE-47	1.3 (<1.0 - 13.6)	Jakobsson et al. 2002
e-waste dismantlers, South-China	BDE-47	9.5 (n.d. - 180)	
	ΣPBDE[b]	600 (140 - 8500)	Bi et al. 2007
foam workers, US	BDE-47	77.8 (19.5 - 540)	
	ΣPBDEs	160 (67 - 973)	Stapleton et al. 2008
14 year-olds working	BDE-47	n.a. (330.5)	
on waste-dump, Nicaragua	ΣPBDEs[b]	n.a. (656.5)	
(pooled samples)	6-OH-BDE-47	n.a. (6.2)	
	ΣOH-PBDE	n.a. (61.5)	Athanasiadou et al. 2008

n.d., not detected; n.a., not applicable; (OH-)PBDE, (hydroxylated) polybrominated diphenyl ethers
[a] for a more extensive review, see Frederiksen et al. 2009, [b] including BDE-209, [c] unit = pg/g ww.

In wildlife as well as human tissues, PBDE congeners BDE-28, BDE-47, BDE-99, BDE-100, BDE-153 and BDE-183 are particularly observed, as well as, although at lower concentrations, BDE-209 (reviewed in Hakk and Letcher 2003). Usually, BDE-47 is the predominant congener in biotic samples (for reviews see Frederiksen et al. 2009; Hites 2004).

PBDEs have been detected in liver, blood, milk and adipose tissues, occasionally at high concentrations, in both wildlife (for reviews see de Wit 2002; Law et al. 2003) and human tissues, including breast milk (Bradman et al. 2007; Petreas et al. 2003; Schecter et al. 2003; Sjödin et al. 2004; reviewed in Frederiksen et al. 2009). Toxicokinetics studies in rodents (Chen et al. 2006; Hakk et al. 2002; Örn and Klasson-Wehler 1998; Sanders et al. 2006a; Staskal et al. 2006; von Meyerinck et al. 1990; reviewed in Darnerud et al. 2001; Hakk and Letcher 2003) demonstrated high absorption and slow elimination as well as accumulation in adipose tissue after a single oral dose of tetra-, penta- and hexaBDEs. Studies in fish showed an efficient absorbance of PBDEs, with a negative correlation with bromination degree (Burreau et al. 1997, 2004). BDE-209 has also been detected in bird's eggs, birds, fish and marine mammals (reviewed in Law et al. 2006) as well as human tissues (reviewed in Frederiksen et al. 2009) despite its poor absorption in the gastrointestinal tract, low solubility, high log octanol-water partition coefficient (K_{ow}) and molecular weight (Mörck et al. 2003). Ecotoxicological concern has arisen from the observation of very high concentrations of BDE-209 in birds of prey in North-China. This observation is a sign of significant biomagnifications of BDE-209 in terrestrial food chains (Chen et al. 2007).

In addition to parent PBDE congeners, hydroxylated and methoxylated PBDEs (OH- and MeO-PBDEs) have also been detected in marine and freshwater fish, sea birds as well as dolphins, seals and polar bears (Gebbink et al. 2008; Houde et al. 2009; Kelly et al. 2008; Kierkegaard et al. 2004; Malmvärn et al. 2005; Marsh et al. 2004; McKinney et al. 2006; Olsson et al. 2000; Routti et al. 2009; Verreault et al. 2005; Wan et al. 2009).

In *in vivo* toxicokinetics studies, OH-PBDEs were detected in liver, lung, plasma, feces and bile after oral administration of BDE-47 or BDE-99 to rats (Chen et al. 2006; Hakk et al. 2002; Marsh et al. 2006; Örn and Klasson-Wehler 1998). OH-PBDEs have also been observed in plasma after intraperitonal administration of an equimolar mixture of environmentally relevant PBDEs to rats (Malmberg et al. 2005). Intravenous administration of BDE-47, BDE-99, BDE-100 or BDE-153 to mice revealed that hydroxylated metabolites were formed from all four PBDEs. BDE-99 was observed to be most readily metabolized by oxidation and oxidation/debromination, while debromination was not observed for the other PBDEs (Staskal et al. 2006). In contrast, metabolism of BDE-153 after oral administration is minimal (Qiu et al. 2007; Sanders et al. 2006b), which is suggested to be due to the absence of Br-atoms with 2 adjacent unsubstituted C-atoms. In support of this explanation, the presence of several OH-PBDEs was observed in feces after oral administration of BDE-154, in which a Br-atom with two unsubstituted adjacent C-atoms is present (Hakk et al. 2009). After oral administration of BDE-209 to rats, several methoxylated and acetylated metabolites were detected in bile and feces (Mörck et al. 2003). In addition, hydroxylated octa- and nonaBDEs were also detected in plasma and the liver after oral or intravenous administration of BDE-209 to rats (Sandholm et al. 2003; Riu et al. 2008). After subchronic low dose administration of DE-71 through the feed to rats, OH-PBDEs were identified in feces (Huwe et al. 2007). In mice, OH-PBDEs were observed in plasma after oral or subcutaneous exposure to DE-71 (Qiu et al. 2007).

Formation of OH-PBDEs from BDE-47 was also demonstrated using phenobarbital-induced rat liver microsomes (Hamers et al. 2008). Recently, the formation of OH-PBDEs was also investigated in human primary hepatocytes exposed to BDE-99 or BDE-209. These cells metabolized BDE-99 into OH-PBDEs while in contrast, OH-PBDEs were not detected after exposure to BDE-209 (Stapleton et al. 2009). Recently, *in vitro* biotransformation of parent, OH- and MeO-PBDEs in rainbow trout, chicken and rat microsomes suggested an additional metabolic pathway, i.e., formation of OH-PBDEs from MeO-PBDEs (Wan et al. 2009).

A distribution study with radiolabeled PBDEs in mice showed that fetal uptake during gestation was relatively limited, while maternal transfer via breast milk resulted in transfer of approximately 20% of the administered dose to the offspring (Darnerud and Risberg 2006). Assuming similar toxicokinetics of PBDEs in humans during gestation and lactation, this suggests that exposure through lactation is also from a quantitative point of view an important exposure route for PBDEs as well as OH-PBDEs in humans (Lacorte and Ikonomou 2009). In fetal liver and placental tissue, CYP enzyme activity is present (Hakkola et al. 1998). Placental transfer of hydroxylated polychlorinated biphenyls (OH-PCBs) has been demonstrated in experimental studies (Meerts et al. 2002). Although placental transfer of OH-PBDEs has not yet been proven, it is not unlikely (especially for lower-brominated PBDEs), due to the structural resemblance with OH-PCBs. Therefore, the internal fetal exposure to OH-PBDEs may be due to fetal hydroxylation and/or placental transfer.

Supplemental Material, References

Athanasiadou M, Cuadra SN, Marsh G, Bergman Å, Jakobsson K. 2008. Polybrominated diphenyl ethers (PBDEs) and bioaccumulative hydroxylated PBDE metabolites in young humans from Managua, Nicaragua. Environ Health Perspect 116:400-408.

Bi X, Qu W, Sheng G, Zhang W, Mai B, Chen D, et al. 2006. Polybrominated diphenyl ethers in South China maternal and fetal blood and breast milk. Environ Pollut 144:1024-1030.

Bi X, Thomas GO, Jones KC, Qu W, Sheng G, Martin FL, et al. 2007. Exposure of electronics dismantling workers to polybrominated diphenyl ethers, polychlorinated biphenyls, and organochlorine pesticides in South China. Environ Sci Technol 41:5647-5653.

Bradman A, Fenster L, Sjödin A, Jones RS, Patterson DG Jr, Eskenazi B. 2007. Polybrominated diphenyl ether levels in the blood of pregnant women living in an agricultural community in California. Environ Health Perspect 115:71-74.

Burreau S, Axelman J, Broman D, Jakobsson E. 1997. Dietary uptake in pike (*Esox lucius*) of some polychlorinated biphenyls, polychlorinated naphtalenes and polybrominated diphenyl ethers administered in natural diet. Environ Toxicol Chem 16:2508-2513.

Burreau S, Zebühr Y, Broman D, Ishaq R. 2004. Biomagnification of polychlorinated biphenyls (PCBs) and polybrominated diphenyl ethers (PBDEs) studied in pike (*Esox lucius*), perch (*Perca fluviatilis*) and roach (*Rutilus rutilus*) from the Baltic Sea. Chemosphere 55:1043-1052.

Chen LJ, Lebetkin EH, Sanders JM, Burka LT. 2006. Metabolism and disposition of 2,2',4,4',5-pentabromodiphenyl ether (BDE99) following a single or repeated administration to rats or mice. Xenobiotica 36:515-534.

Chen D, Mai B, Song J, Sun Q, Luo Y, Luo X, et al. 2007. Polybrominated diphenyl ethers in birds of prey from Northern China. Environ Sci Technol 41:1828-1833.

Darnerud PO, Eriksen GS, Jóhannesson T, Larsen PB, Viluksela M. 2001. Polybrominated diphenyl ethers: occurrence, dietary exposure, and toxicology. Environ Health Perspect 109 Suppl 1:49-68.

Darnerud PO, Risberg S. 2006. Tissue localisation of tetra- and pentabromodiphenyl ether congeners (BDE-47, -85 and -99) in perinatal and adult C57BL mice. Chemosphere 62:485-493.

de Wit CA. 2002. An overview of brominated flame retardants in the environment. Chemosphere 46:583-624.

Fischer D, Hooper K, Athanasiadou M, Athanassiadis I, Bergman Å. 2006. Children show highest levels of polybrominated diphenyl ethers in a California family of four: a case study. Environ Health Perspect 114:1581-1584.

Frederiksen M, Vorkamp K, Thomsen M, Knudsen LE. 2009. Human internal and external exposure to PBDEs--a review of levels and sources. Int J Hyg Environ Health 212:109-134.

Gebbink WA, Sonne C, Dietz R, Kirkegaard M, Riget FF, Born EW, et al. 2008. Tissue-specific congener composition of organohalogen and metabolite contaminants in East Greenland polar bears (*Ursus maritimus*). Environ Pollut 152:621-629.

Gómara B, Herrero L, Ramos JJ, Mateo JR, Fernández MA, García JF, et al. 2007. Distribution of polybrominated diphenyl ethers in human umbilical cord serum, paternal serum, maternal serum, placentas, and breast milk from Madrid population, Spain. Environ Sci Technol 41:6961-6968.

Guvenius DM, Aronsson A, Ekman-Ordeberg G, Bergman Å, Norén K. 2003. Human prenatal and postnatal exposure to polybrominated diphenyl ethers, polychlorinated biphenyls, polychlorobiphenylols, and pentachlorophenol. Environ Health Perspect 111:1235-1241.

Hakk H, Huwe JK, Larsen GL. 2009. Absorption, distribution, metabolism and excretion (ADME) study with 2,2',4,4',5,6'-hexabromodiphenyl ether (BDE-154) in male Sprague-Dawley rats. Xenobiotica 39:46-56.

Hakk H, Larsen G, Klasson-Wehler E. 2002. Tissue disposition, excretion and metabolism of 2,2',4,4',5-pentabromodiphenyl ether (BDE-99) in the male Sprague-Dawley rat. Xenobiotica 32:369-382.

Hakk H, Letcher RJ. 2003. Metabolism in the toxicokinetics and fate of brominated flame retardants--a review. Environ Int 29:801-828.

Hakkola J, Pelkonen O, Pasanen M, Raunio H. 1998. Xenobiotic-metabolizing cytochrome P450 enzymes in the human feto-placental unit: role in intrauterine toxicity. Crit Rev Toxicol 28:35-72.

Hamers T, Kamstra JH, Sonneveld E, Murk AJ, Visser TJ, Van Velzen MJ, et al. 2008. Biotransformation of brominated flame retardants into potentially endocrine-disrupting metabolites, with special attention to 2,2',4,4'-tetrabromodiphenyl ether (BDE-47). Mol Nutr Food Res 52:284-298.

Hites RA. 2004. Polybrominated diphenyl ethers in the environment and in people: a meta-analysis of concentrations. Environ Sci Technol 38:945-956.

Houde M, Pacepavicius G, Darling C, Fair PA, Alaee M, Bossart GD, et al. 2009. Polybrominated diphenyl ethers and their hydroxylated analogs in plasma of bottlenose dolphins (*Tursiops truncatus*) from the United States East Coast. Environ Toxicol Chem 28:2061-2068.

Huwe J, Hakk H, Lorentzsen M. 2007. Bioavailability and mass balance studies of a commercial pentabromodiphenyl ether mixture in male Sprague-Dawley rats. Chemosphere 66:259-266.

Jakobsson K, Thuresson K, Rylander L, Sjödin A, Hagmar L, Bergman Å. 2002. Exposure to polybrominated diphenyl ethers and tetrabromobisphenol A among computer technicians. Chemosphere 46:709-716.

Karlsson M, Julander A, van Bavel B, Hardell L. 2007. Levels of brominated flame retardants in blood in relation to levels in household air and dust. Environ Int 33:62-69.

Kelly BC, Ikonomou MG, Blair JD, Gobas FAPC. 2008. Hydroxylated and methoxylated polybrominated diphenyl ethers in a Canadian Arctic marine food web. Environ Sci Technol 42:7069-7077.

Kierkegaard A, Bignert A, Sellström U, Olsson M, Asplund L, Jansson B, et al. 2004. Polybrominated diphenyl ethers (PBDEs) and their methoxylated derivatives in pike from Swedish waters with emphasis on temporal trends, 1967-2000. Environ Pollut 130:187-198.

Lacorte S, Ikonomou MG. 2009. Occurrence and congener specific profiles of polybrominated diphenyl ethers and their hydroxylated and methoxylated derivatives in breast milk from Catalonia. Chemosphere 74:412-420.

Law RJ, Alaee M, Allchin CR, Boon JP, Lebeuf M, Lepom P, et al. 2003. Levels and trends of polybrominated diphenylethers and other brominated flame retardants in wildlife. Environ Int 29:757-770.

Law RJ, Allchin CR, de Boer J, Covaci A, Herzke D, Lepom P, et al. 2006. Levels and trends of brominated flame retardants in the European environment. Chemosphere 64:187-208.

Malmberg T, Athanasiadou M, Marsh G, Brandt I, Bergman Å. 2005. Identification of hydroxylated polybrominated diphenyl ether metabolites in blood plasma from polybrominated diphenyl ether exposed rats. Environ Sci Technol 39:5342-5348.

Malmvärn A, Marsh G, Kautsky L, Athanasiadou M, Bergman Å, Asplund L. 2005. Hydroxylated and methoxylated brominated diphenyl ethers in the red algae *Ceramium tenuicorne* and blue mussels from the Baltic Sea. Environ Sci Technol 39:2990-2997.

Marsh G, Athanasiadou M, Athanassiadis I, Sandholm A. 2006. Identification of hydroxylated metabolites in 2,2',4,4'-tetrabromodiphenyl ether exposed rats. Chemosphere 63:690-697.

Marsh G, Athanasiadou M, Bergman Å, Asplund L. 2004. Identification of hydroxylated and methoxylated polybrominated diphenyl ethers in Baltic Sea salmon (*Salmo salar*) blood. Environ Sci Technol 38:10-18.

Mazdai A, Dodder NG, Abernathy MP, Hites RA, Bigsby RM. 2003. Polybrominated diphenyl ethers in maternal and fetal blood samples. Environ Health Perspect 111:1249-1252.

McKinney MA, Cesh LS, Elliott JE, Williams TD, Garcelon DK, Letcher RJ. 2006. Brominated flame retardants and halogenated phenolic compounds in North American west coast bald eaglet (*Haliaeetus leucocephalus*) plasma. Environ Sci Technol 40:6275-6281.

Meerts IATM, Assink Y, Cenijn PH, van den Berg JHJ, Weijers BM, Bergman Å, et al. 2002. Placental transfer of a hydroxylated polychlorinated biphenyl and effects on fetal and maternal thyroid hormone homeostasis in the rat. Toxicol Sci 68:361-371.

Meijer L, Weiss J, van Velzen M, Brouwer A, Bergman Å, Sauer PJJ. 2008. Serum concentrations of neutral and phenolic organohalogens in pregnant women and some of their infants in The Netherlands. Environ Sci Technol 42:3428-3433.

Mörck A, Hakk H, Örn U, Klasson-Wehler E. 2003. Decabromodiphenyl ether in the rat: absorption, distribution, metabolism, and excretion. Drug Metab Dispos 31:900-907.

Olsson A, Ceder K, Bergman Å, Helander B. 2000. Nestling blood of the white-tailed sea eagle (*Haliaeetus albicilla*) as an indicator of territorial exposure to organohalogen compounds−an evaluation. Environ Sci Technol 34:2733–2740.

Örn U, Klasson-Wehler E. 1998. Metabolism of 2,2',4,4'-tetrabromodiphenyl ether in rat and mouse. Xenobiotica 28:199-211.

Petreas M, She J, Brown FR, Winkler J, Windham G, Rogers E, et al. 2003. High body burdens of 2,2',4,4'-tetrabromodiphenyl ether (BDE-47) in California women. Environ Health Perspect 111:1175-1179.

Qiu X, Bigsby RM, Hites RA. 2009. Hydroxylated metabolites of polybrominated diphenyl ethers in human blood samples from the United States. Environ Health Perspect 117:93-98.

Qiu X, Mercado-Feliciano M, Bigsby RM, Hites RA. 2007. Measurement of polybrominated diphenyl ethers and metabolites in mouse plasma after exposure to a commercial pentabromodiphenyl ether mixture. Environ Health Perspect 115:1052-1058.

Ramos JJ, Gómara B, Fernández MA, González MJ. 2007. A simple and fast method for the simultaneous determination of polychlorinated biphenyls and polybrominated diphenyl ethers in small volumes of human serum. J Chromatogr A 1152:124-129.

Riu A, Cravedi JP, Debrauwer L, Garcia A, Canlet C, Jouanin I, et al. 2008. Disposition and metabolic profiling of [^{14}C]-decabromodiphenyl ether in pregnant Wistar rats. Environ Int 34:318-329.

Routti H, Letcher RJ, Chu S, van Bavel B, Gabrielsen GW. 2009. Polybrominated diphenyl ethers and their hydroxylated analogues in ringed seals (*Phoca hispida*) from Svalbard and the Baltic Sea. Environ Sci Technol 43:3494-3499.

Sanders JM, Chen LJ, Lebetkin EH, Burka LT. 2006a. Metabolism and disposition of 2,2',4,4'- tetrabromodiphenyl ether following administration of single or multiple doses to rats and mice. Xenobiotica 36:103-117.

Sanders JM, Lebetkin EH, Chen LJ, Burka LT. 2006b. Disposition of 2,2',4,4',5,5'-hexabromodiphenyl ether (BDE153) and its interaction with other polybrominated diphenyl ethers (PBDEs) in rodents. Xenobiotica 36:824-837.

Sandholm A, Emanuelsson BM, Wehler EK. 2003. Bioavailability and half-life of decabromodiphenyl ether (BDE-209) in rat. Xenobiotica 33:1149-1158.

Schecter A, Pavuk M, Päpke O, Ryan JJ, Birnbaum L, Rosen R. 2003. Polybrominated diphenyl ethers (PBDEs) in U.S. mothers' milk. Environ Health Perspect 111:1723-1729.

Sjödin A, Hagmar L, Klasson-Wehler E, Kronholm-Diab K, Jakobsson E, Bergman Å. 1999. Flame retardant exposure: polybrominated diphenyl ethers in blood from Swedish workers. Environ Health Perspect 107:643-648.

Sjödin A, Jones RS, Focant JF, Lapeza C, Wang RY, McGahee EE 3rd, et al. 2004. Retrospective time-trend study of polybrominated diphenyl ether and polybrominated and polychlorinated biphenyl levels in human serum from the United States. Environ Health Perspect 112:654-658.

Stapleton HM, Kelly SM, Pei R, Letcher RJ, Gunsch C. 2009. Metabolism of polybrominated diphenyl ethers (PBDEs) by human hepatocytes *in vitro*. Environ Health Perspect 117:197-202.

Stapleton HM, Sjödin A, Jones RS, Niehüser S, Zhang Y, Patterson DG Jr. 2008. Serum levels of polybrominated diphenyl ethers (PBDEs) in foam recyclers and carpet installers working in the United States. Environ Sci Technol 42:3453-3458.

Staskal DF, Hakk H, Bauer D, Diliberto JJ, Birnbaum LS. 2006. Toxicokinetics of polybrominated diphenyl ether congeners 47, 99, 100, and 153 in mice. Toxicol Sci 94:28-37.

Thomsen C, Lundanes E, Becher G. 2001. Brominated flame retardants in plasma samples from three different occupational groups in Norway. J Environ Monit 3:366-370.

Toms LML, Sjödin A, Harden F, Hobson P, Jones R, Edenfield E, et al. 2009. Serum polybrominated diphenyl ether (PBDE) levels are higher in children (2-5 years of age) than in infants and adults. Environ Health Perspect 117:1461-1465.

Verreault J, Gabrielsen GW, Chu S, Muir DCG, Andersen M, Hamaed A, et al. 2005. Flame retardants and methoxylated and hydroxylated polybrominated diphenyl ethers in two Norwegian Arctic top predators: glaucous gulls and polar bears. Environ Sci Technol 39:6021-6028.

von Meyerinck L, Hufnagel B, Schmoldt A, Benthe HF. 1990. Induction of rat liver microsomal cytochrome P-450 by the pentabromo diphenyl ether Bromkal 70 and half-lives of its components in the adipose tissue. Toxicology 61:259-274.

Wan Y, Choi K, Kim S, Ji K, Chang H, Wiseman S, et al. 2010. Hydroxylated polybrominated diphenyl ethers and bisphenol A in pregnant women and their matching fetuses: placental transfer and potential risks. Environ Sci Technol 44:5233-5239.

Wan Y, Wiseman S, Chang H, Zhang X, Jones PD, Hecker M, et al. 2009. Origin of hydroxylated brominated diphenyl ethers: natural compounds or man-made flame retardants. Environ Sci Technol 43:7536-7542.
